# Microcornea, iris and choroidal coloboma, and global developmental delay caused by *TENM3* pathogenic variants in a Chinese patient

**DOI:** 10.1002/mgg3.1948

**Published:** 2022-04-09

**Authors:** Youfeng Zhou, Ke Xu, Weiyue Gu, Yan Huang

**Affiliations:** ^1^ Department of Pediatrics Fujian Provincial Maternity and Children's Hospital, Affiliated Hospital of Fujian Medical University Fuzhou China; ^2^ Beijing Chigene Translational Medicine Research Center Co., Ltd Beijing China; ^3^ Child Healthcare Department Fujian Provincial Maternity and Children's Hospital, Affiliated Hospital of Fujian Medical University Fuzhou China

**Keywords:** congenital heart defects, global developmental delay, Microphthalmia, *TENM3*

## Abstract

**Background:**

Biallelic *TENM3* pathogenic variants cause isolated or syndromic microphthalmia. Syndromic microphthalmia 15 (MCOPS15) is characterized by microphthalmia, coloboma, and developmental delay. Currently, only four cases of MCOPS15 have been reported and the clinical features varied among the patients indicating potential broad phenotypic spectrum.

**Methods:**

The present case was a 6‐month‐old male at diagnosis. The patient exhibited long philtrum, large ears, bilateral ptosis, and nystagmus. Ophthalmic tests showed that he had microcornea, iris and choroidal coloboma. The patient presented with global developmental delay (GDD). Trio‐whole exome sequencing and genome copy number sequencing were conducted to explore the disease‐causing mutations.

**Results:**

Exome sequencing and genome copy number sequencing showed the presence of L1471F and E661G compound mutations in *TENM3*, which were inherited from the mother and father, respectively. Sanger sequencing was conducted to verify association of the mutations with the disease in the present family.

**Conclusion:**

Two *TENM3* variants were identified in a patient with Syndromic microphthalmia 15 in the present study. However, further studies should be conducted to explore the pathogenicity of the variants.

## Graphical abstract

To date, only four cases of MCOPS15 were reported and the clinical features of the patients were variable, which indicate potential broad phenotypic spectrum. In this study, using trio‐whole exome sequencing, we identified biallelic variants (L1471F and E661G) of TENM3, in a 6 months Chinese boy. Our findings expand the mutational spectrum of the TENM3 gene, which might help increases understanding of the heterogeneity of clinical manifestations of diseases.

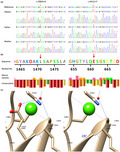

## 1 INTRODUCTION

Syndromic microphthalmia 15 (MCOPS15, MIM: #615145) is an extremely rare disease caused by homozygous or compound pathogenic variants of the *TENM3* gene. Currently, only four cases of MCOPS15 have been previously reported and the clinical features of the patients varied, indicating potential broad phenotypic spectrum (Chassaing et al., 2016; Singh et al., 2019; Stephen et al., 2018). The previous cases presented with microphthalmia, hence the name of the disease. The genotype–phenotype correlation of MCOPS15 has not been fully elucidated and the reported cases showed distinct phenotype associated with *TENM3* variants without specific biological pathogenicity. Previous studies report high genotype and phenotype variation in MCOPS15 patients.

## 2 MATERIALS AND METHODS

### 2.1 Patient

**FIGURE 1:**
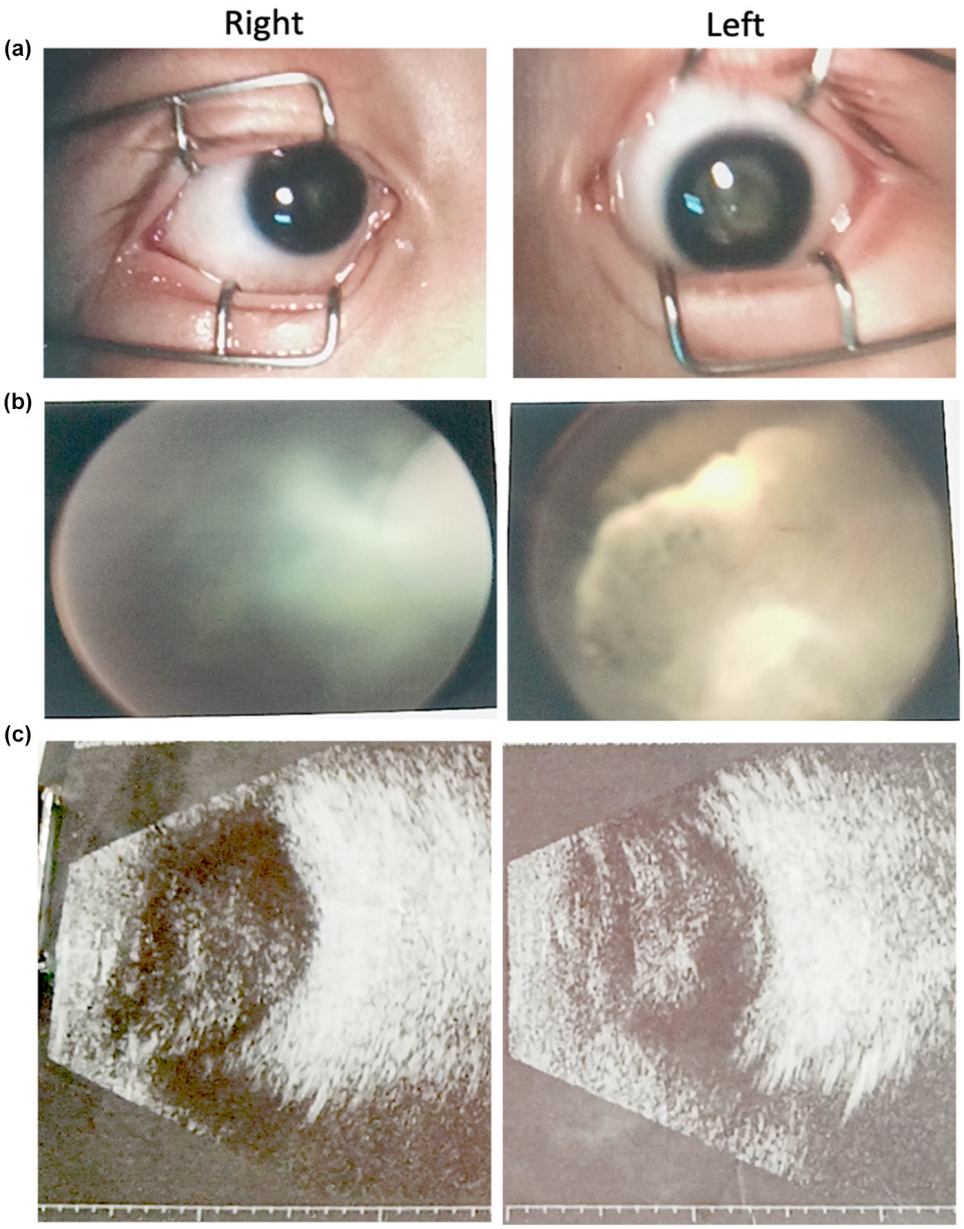
Eye examination in the patient. (a) Iris coloboma. (b) Ophthalmoscope shows the choroidal coloboma. (c) Ophthalmic ultrasound showed vitreous opacity in both eyes and left retinal detachment

The patient in the present study was a 1‐year‐old male who was previously presented in our hospital at 6 months of age due to acute respiratory infection. The patient was the second child born to a non‐consanguineous couple without family history of congenital diseases. Perinatal and prenatal examinations were normal, and there was no history of exposure to toxic substances. The height and weight of the patient were within the normal range for children of the same age. He underwent normal breast feeding and received infant feeding. The patient had no history of previously unexpected or abnormal treatment. The patient presented with long philtrum, large ears, bilateral ptosis, and nystagmus (his guardian did not allow publication of the photos) and mild hypertonia of lower limbs. The patient could not crawl or sit without assistance at 1 year of age. In addition, his cognitive and emotional responses were severely impaired, indicating possible severe global developmental delay (GDD). The patient underwent the full program of developmental assessment because he was not cooperating during the tests. Ophthalmic tests showed that he had microcornea, iris and choroidal coloboma (Figure 1a,b). Furthermore, the patient had bilateral NLP (no light perception) at age 1 and the follow‐up examination showed significant iris and choroidal coloboma (Figure 1c).

Blood and biochemical tests at the time of admission showed WBC (white blood cell) count of 7.13 × 10^9^/L, elevated NE (neutrophilic granulocyte) ratio of 47.2%, platelet count of 384 × 10^9^/L, and CRP (C‐reactive protein) level of 12.79 mg/L. Liver and kidney function, enzyme, and metabolic tests of the patient were normal. His urine and stool tests were normal.

**FIGURE 2:**
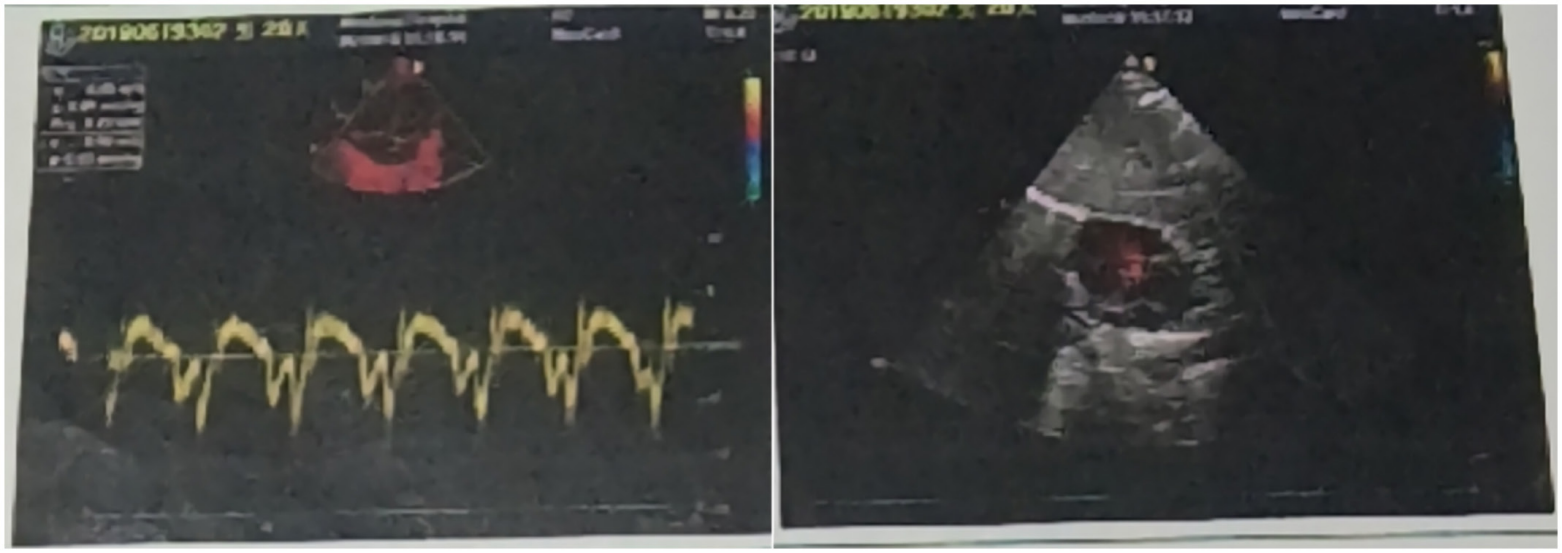
Doppler ultrasound showed multiple heart defects in the patient

Doppler ultrasound showed that the patient had congenital heart defects (CHDs) including: atrial septal defect, patent foramen ovale, patent ductus arteriosus, mildly dilated right atrium and ventricle, mild right ventricular hypertrophy, mild tricuspid valve regurgitation, and pulmonary arterial hypertension (Figure 2). EEG and brain MRI examination of the patient were normal.

Differential diagnosis was conducted to review diseases of syndromic ocular coloboma with CHDs. The reviewing criteria were retrieved from the Online Mendelian Inheritance in Man (OMIM) database and previous research articles (Chang et al., 2006; Lingam et al., 2021; Prichard, 1992).

Furthermore, a comparison of clinical characteristics in the present patient and previously reported MCOPS15 cases was performed (Table 2).

The patient was discharged at 1 year of age.

### 2.2 Genetic tests and in silico analysis

Genomic DNA was extracted from peripheral blood of the triad and whole exome sequencing (WES) and copy number variation sequencing (CNV‐seq) were performed (Xie & Tammi, 2009). The libraries for WES were prepared using the xGen Exome Research Panel v1.0 (IDT, USA) kit, and sequencing was conducted on a NovaSeq 6000 platform (Illumina, San Diego, CA, USA). CNV‐seq was performed as previously described (Xie & Tammi, 2009). Sequences were aligned using VarMap (https://www.ebi.ac.uk/thornton‐srv/databases/cgi‐bin/VarSite/GetPage.pl?varmap=TRUE), an online protein structural annotation tool hosted by EMBL's European Bioinformatics Institute (EMBL‐EBI). The three‐dimensional protein structure of TENM3 has not been experimentally solved, therefore, the homolog structure of notch1 (pdb id: 5uk5), a *Rattus norvegicus* protein was used, and E661 was matched to E352 in the PDB structure.

## 3 RESULTS

**FIGURE 3:**
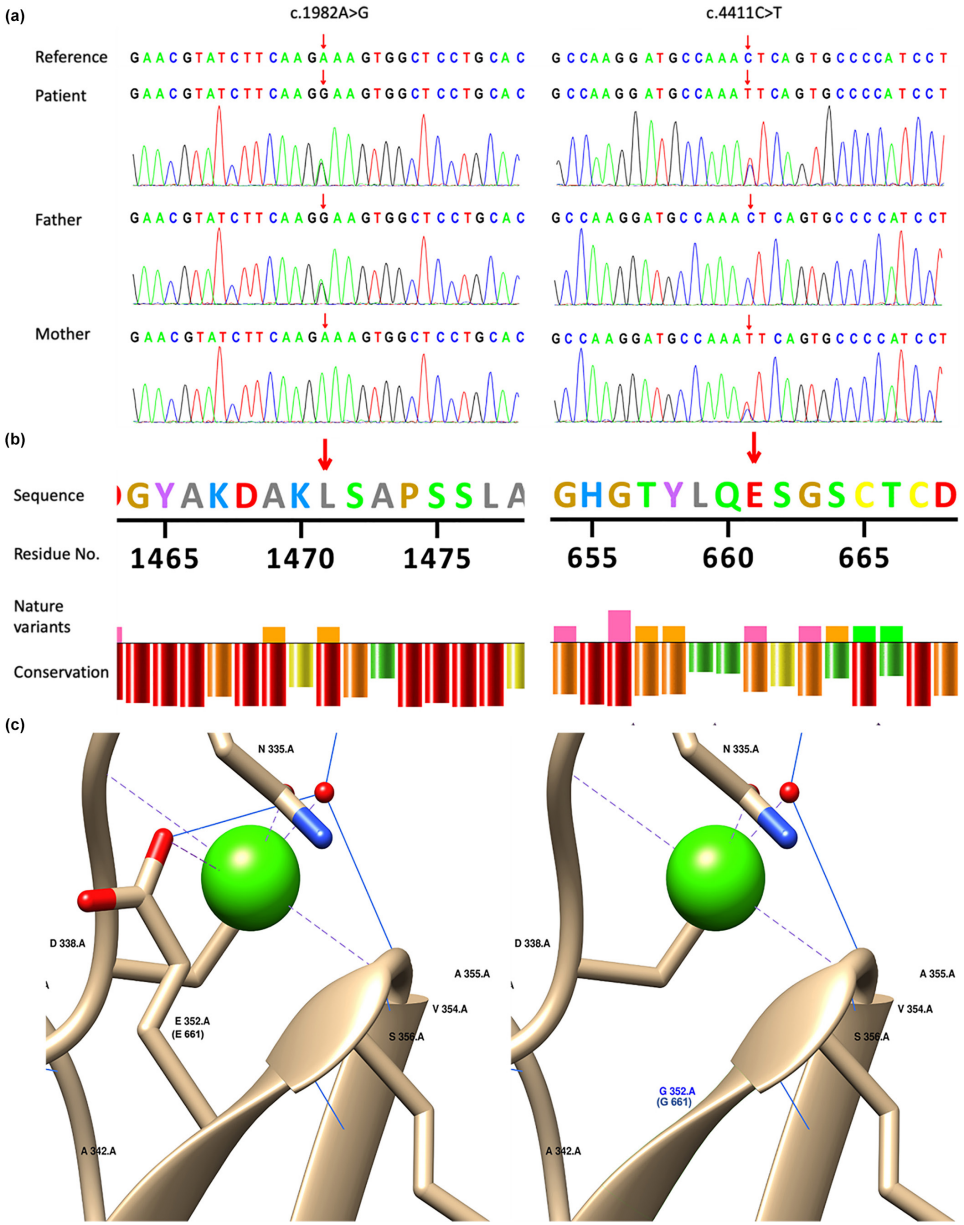
(a) Sanger sequencing confirm *TENM3* variants in the patient and his parents. (b) Conservation of L1471 and E661, generated by VarMap, EMBL‐EBI. Bar colors green, yellow, orange, and red indicate the low, medium, high, and highest conservation across species, respectively. (c) Three‐dimensional demonstration of E661G, mapped to E352G (pdb id: 5uk5). The green ball shows calcium ion, dashed lines in purple show protein–metal interaction

The findings showed the presence of the compound *TENM3* variants, NM_001080477.3: c.4411C > T (chr4:183675931C > T, hg19, p.L1471F, dbSNP: rs569985378) and c.1982A > G (chr4: 183603114A > G, hg19, p.E661G, dbSNP: rs766092012) in the patient and the variants were further verified through Sanger sequencing (Figure 3a). Sanger sequencing was performed using primers F‐ATCAGTTGGAGACGCAACTTCATA, R‐AAAATTAACCGGATAAGGCAGGTC, and F‐TCATGGTGTGTGTATCCACGG, R‐AACTGGCTAAGCCTGGTTCCT for c.4411 and c.1982 sequencing, respectively. CNV‐seq showed no disease‐causing CNV. Variants L1471F and E661G have been previously documented only in Asian population with minor allele frequencies (MAFs) of 0.023% in 48,574 samples, and 0.021% in 48,570 samples (gnomAD), respectively. Pathogenicity of the two variants was reported as “variant of uncertain significance” according to the American College of Medical Genetics (ACMG) clinical guidelines with evidence PP3 (several computational studies support a deleterious effect). L1471F was predicted as damaging or possibly damaging using Provean, SIFT, Polyphen2, mutationtaster, M‐CAP, and REVEL tools. Analysis using Provean, SIFT, and mutationtaster tools indicated that E661G was deleterious. Multiple sequence alignment results showed a high conservation of L1471 (Figure 3b). Structural analysis indicated that E352G (E661G) disrupts the protein–metal interaction in the at the active site of the protein (Figure 3c). Analysis of L1471F after homology modeling showed no potential intuitive damage. In addition, no pathogenic variation in the genes listed in Table 1 or in other disease‐associated genes and chromosome regions were associated with the patient phenotype.

**TABLE 1:** Inherited diseases with congenital heart disease and iris coloboma with or without choroidal coloboma for differential diagnosis in this study

Diseases	OMIM ID	Systemic phenotypes	Genetic locus
Kapur–Toriello syndrome	% 244300	CHD, cleft lip, cleft palate, distinctive nose	Unknown
Chime syndrome	#280000	CHD, migratory ichthyosiform dermatosis, ID, deafness	*PIGL*
Townes Brocks syndrome 1	#107480	CHD, imperforate anus, polydactyly, triphalyngeal thumb, dysplastic ears, renal abnormalities	*SALL1*
Steinfeld syndrome	184705	CHD, cleft palate, holoprosencephaly, dysplastic ears, vertebral anomalies, kidney malformations	Unknown
Kabuki syndrome 1	#147920	CHD, facial deformation: long palpebral fissures, prominent eyelashes, arched eyebrows with lateral thinning, epicanthus, eversion of the lateral third of the lower eyelid, renal defects	*KMT2D*
Syndromic microphthalmia with CHD9	#309800, #601186, #615877, #206900	CHD, ID, palatal and dental anomalies, renal defects	*NAA10*, *STRA6*, *MAB21L2*, *SOX2*

Abbreviations: CHD, congenital heart disease; ID, intellectual disability.

**TABLE 2:** Clinical characteristic comparison of the patient and previously reported patients diagnosed with MCOPS15

	Patient 1 (Aldahmesh et al., 2012)	Patient 2 (Aldahmesh et al., 2012)	Patient 3 (Chassaing et al., 2016)	Patient 4 (Stephen et al., 2018)	Patient 5 (Stephen et al., 2018)	Patient 6 (Singh et al., 2019)	Patient 7 (Maddirevula et al., 2020)	Present study
Age at diagnosis	11 years	9 years	9 years	5 years 6 months	4 years 3 months	6 years		6 months
Sex	Male	Female	Male	Female	Female	Male	Female	Male
Consanguinity	Yes	Yes	Yes	No	No	No	NA	No
Facial abnormality	NA	NA	NA	Broad eyebrows, hypertelorism, narrow palpebral fissures, long philtrum, low‐set flared pinnae	Broad eyebrows, hypertelorism, narrow palpebral fissures, long philtrum, low‐set flared pinnae	Plagiocephaly, low anterior hairline, supraorbital flattening, large ears	NA	Long philtrum, large ears, nystagmus
Microphthalmia	Present	Present	Present	Absent	Absent	Present	Present	Absent
ACM	NA	NA	NA	NA	NA	NA	NA	Disappear (left), shallow (right)
Microcornea	Present	Present	Present	Present	Present	Bilateral sclerocornea	Present	Present
Corneal shape	NA	NA	NA	Vertically oval	Vertically oval	NA	NA	Round
Pupils	NA	NA	NA	Keyhole shaped	Keyhole shaped	NA	NA	Irregular
Iris coloboma	Present	Present	Inferior	Inferonasal	Inferonasal	NA	NA	Inferior
Fundus coloboma	Severe discs and macula coloboma	Severe discs and macula coloboma						choroidal coloboma
VO	NA	NA	NA	NA	NA	NA	NA	Present
Cataract	NA	NA	NA	NA	NA	NA	NA	Present
VA	20/50 left eye, HM right eye	20/200 right eye, 20/300 left eye	HM both eyes	6/36 both eyes	6/36 both eyes		NA	NLP
Ptosis	NA	NA	Absent	Unilateral (left)	Bilateral (partial)	Absent	NA	Bilateral
Development	Normal	Normal	GDD	GDD	MDD	GDD	NA	GDD with hypertonia
Pathogenic variants	Hom. c.2083dup/p.T695Nfs*5	Hom. c.2083dup/p.T695Nfs*5	Hom. c.2968‐2A > T/p.V990Cfs*13	Hom. c.1857 T > A/p. C619*	Hom. c.1857 T > A/p. C619*	c.7687C > T/p. R2563W and c.4046C > G; p. A1349G	Hom. c.6006_6009del/p.Gln2003Phefs*10	c.4411C > T p.L1471F and c.1982A > G/p.E661G

Abbreviations: ACM, anterior chamber malformation; GDD, global developmental delay; HM, hand movement; Hom., homozygous; MDD, motor developmental delay; NA, not available; NLP, no light perception; TR, tricuspid insufficiency; VA, visual acuity; VO, vitreous opacities.

## 4 DISCUSSION

Facial deformation, ocular coloboma, and central nervous impairment symptoms observed in the present patient are consistent with syndromic microphthalmia 15 (MCOPS15) diagnosis. However, microphthalmia was not observed. Stephen et al.(Stephen et al., 2018) reported cases of two sisters presenting with DD and ocular coloboma, without microphthalmia (Table 1), which is consistent with the findings that microphthalmia is not mandatory for MCOPS15 diagnosis. This may be attributed to the young age (<5 years old) at the time diagnosis and the microphthalmia was not significant in the present and previously reported cases (Stephen et al., 2018). Microcornea, iris coloboma, and visual impairment were observed in the present and previous cases, indicating the core phenotype of MCOPS15 disease. Fundus/choroidal coloboma was reported in a study by Aldahmesh et al. (Aldahmesh et al., 2012) and the present study, indicating that fundus/choroidal coloboma is a key symptom in MCOPS15 cases. Occurrence of global or motor developmental delay is a common feature in MCOPS15 patients. However, normal development was observed in the previously siblings at age 9 and 11 years (Aldahmesh et al., 2012). In addition, long philtrum and large ear are common facial deformation in previously reported cases (Singh et al., 2019; Stephen et al., 2018) and the present case. The unexplained CHDs observed in the current case may be attributed to nonhereditary perinatal abnormalities, based on the genetic tests and the absence of CHD family history.

Presence CHDs and coloboma in the same patient can prompt to diagnosis of several diseases (Table 1). The genetic factors of Kapur–Toriello syndrome (MIM: % 244300) and Steinfeld syndrome (MIM: 184705) have not been fully elucidated, reducing effectiveness of the genetic tests (gene panel sequencing) and potentially increasing medical costs. Therefore, affordable, comprehensive sequencing methods including, WES and CNV‐seq, were applied in the current study.

Pathogenicity of the two *TENM3* variants, L1471F and E661G, in the present case has not been reported previously. In addition, pathogenicity of R2563W and A1349G variants reported in a previous study has not been fully elucidated (Singh et al., 2019). This is partly attributed to lack comprehensive analysis of TENM3 protein. In silico analysis using the 3D structure of the protein showed that E661 is located in a calcium‐binding EGF domain and E661G disrupts the protein–metal interaction at this site. The calcium‐binding EGF domain is an EGF‐like domain of approximately 40 amino‐acid residues initially reported in EGF. Calcium ion binding plays a key role in most of the calcium‐binding EGF‐containing proteins (Appella et al., 1988; Davis, 1990; Doolittle et al., 1984). In human, TENM3 is encoded by the *TENM3* (also as known as *ODZ3*, located on chromosome 4q) gene and is expressed in interconnected areas of the developing nervous system and nonneural tissues (Levine et al., 1994; Minet et al., 1999). In mice embryo, Odz3 expression has been reported in the nervous system and mesoderm‐derived tissues (Ben‐Zur et al., 2000). Glendining et al. reported that deletion of Odz3 causes significant disruption of binocular visual alignment and function, indicating downstream changes in axonal guidance (Glendining et al., 2017). Moreover, Berns et al. demonstrated that topographic circuit assembly in mouse hippocampus is regulated by Odz3, implying that Odz3 plays an important role in development of the complex distributed circuit in the mammalian brain (Berns et al., 2018). However, these findings do not explain human developmental disorder associated with TENM3 defects. Therefore, further studies should be conducted to explore the protein structure and effects of TENM3 variants.

Lack of information on the pathogenicity of *TENM3* L1471F and E661G may be a potential cause of the low prevalence of *TENM3* disease. In the current study, diagnosis of MCOPS15 was not originally considered due to the uncertain significance of the variants and the absence of microphthalmia, until the other genetic and nongenetic factors were excluded through differential diagnosis. In addition, the presence of CHDs in the patient complicates differential diagnosis. Notably, L1471F and E661G have only been documented among Asian population with relatively high MAFs (0.023% and 0.021%, respectively) indicating potentially higher risk of *TENM3* disease in China. Currently, Chinese ophthalmologists lack the skills for genetic tests required for accurate diagnosis of the disease.

## 5 CONCLUSION

The *TENM3* L1471F and E661G variants were identified in a Chinese patient with MCOPS15. The findings of the current study showed a relatively high MAFs of these variants in East‐Asian population and the potential absence of neurological disorder, implying that *TENM3* disease may be underreported in China and other East‐Asian countries.

## CONFLICT OF INTEREST

The authors declare that the research was conducted in the absence of any commercial and financial relationships that could be construed as a potential conflict of interest.

## AUTHOR CONTRIBUTIONS

Youfeng Zhou was involved in writing—original draft, investigation, and resources. Ke Xu was involved in formal analysis and visualization. Weiyue Gu was involved in formal analysis, visualization, and writing—review and editing. Yan Huang was involved in conceptualization, resources, methodology, writing—review and editing, funding acquisition, and project administration.

## ETHICS STATEMENT

The study was reviewed and approved by the Ethics Committee of Fujian Provincial Maternity and Children's Hospital. Written informed consent to participate in this study was provided by the participants’ legal guardian.

## Data Availability

The variants have been submitted to the NCBI ClinVar database (Accession number: SCV001911503 and SCV001911504). The data that support the findings of this study are available on request from the corresponding author. The data are not publicly available due to privacy or ethical restrictions.
